# Safety and feasibility of intranasal heroin-assisted treatment: 4-week preliminary findings from a Swiss multicentre observational study

**DOI:** 10.1186/s12954-023-00731-y

**Published:** 2023-01-07

**Authors:** Marc Vogel, Maximilian Meyer, Jean N. Westenberg, Adrian Kormann, Olivier Simon, Roba Salim Hassan Fadlelseed, Markus Kurmann, Rebecca Bröer, Nathalie Devaud, Ulrike Sanwald, Sophie Baumgartner, Hannes Binder, Johannes Strasser, R. Michael Krausz, Thilo Beck, Kenneth M. Dürsteler, Luis Falcato

**Affiliations:** 1 University of Basel Psychiatric Clinics, Wilhelm Klein-Strasse 27, 4002 Basel, Switzerland; 2 Department of Psychiatry, Faculty of Medicine, University of British Columbia, Vancouver, BC Canada; 3 ZOPA Zuger Opiat-Abgabe, Baar, Switzerland; 4 Service of Addiction Medicine, Lausanne University Hospital (CHUV), University of Lausanne, Lausanne, Switzerland; 5 Clinic for Psychiatry and Psychotherapy, Hospitals Schaffhausen, Schaffhausen, Switzerland; 6 HeGeBe HEROL, Psychiatric Services, Hospitals Solothurn, Olten, Switzerland; 7 SuGeBe Gourrama, Psychiatric Services, Hospitals Solothurn, Solothurn, Switzerland; 8 Crossline Clinic, Zurich, Switzerland; 9 Integrierte Suchthilfe Winterthur ISW, Integrated Psychiatry Winterthur, Winterthur, Switzerland; 10 Heroingestützte Behandlung KODA, Bern, Switzerland; 11 Outpatient Clinic for Substance Use Disorders, Psychiatric Clinic Baselland, Reinach, Switzerland; 12 Arud Zentrum Für Suchtmedizin, Zurich, Switzerland; 13 Department of Psychiatry, Psychotherapy and Psychosomatics, Psychiatric Hospital, University of Zurich, Zurich, Switzerland

**Keywords:** Diacetylmorphine, Diamorphine, Heroin-assisted treatment, Substitution, Route of administration, Opioid agonist treatment, Intranasal

## Abstract

**Background:**

Heroin-assisted treatment (HAT) is effective for individuals with severe opioid use disorder (OUD) who do not respond sufficiently to other opioid agonist treatments. It is mostly offered with injectable diacetylmorphine (DAM) or DAM tablets creating a barrier for individuals who need the rapid onset of action but are either unable or unwilling to inject, or primarily snort opioids. To explore another route of administration, we evaluated the safety and feasibility of intranasal (IN) DAM.

**Methods:**

This is a multicentre observational cohort study among patients in Swiss HAT. All patients planning to receive IN DAM within the treatment centres were eligible to participate. Participants were either completely switched to IN DAM or received IN DAM in addition to other DAM formulations or opioid agonists. Patients were followed up for four weeks. Sociodemographic characteristics, current HAT regimen, reasons for starting IN DAM, IN DAM doses, number of injection events in the sample, IN DAM continuation rate, and appearance of adverse events and nose-related problems were evaluated.

**Results:**

Participants (*n* = 52) reported vein damage, preference for nasal route of administration, and desire of a stronger effect or for a less harmful route of administration as primary reasons for switching to IN DAM. After four weeks, 90.4% of participants (*n* = 47) still received IN DAM. Weekly average realised injection events decreased by 44.4% from the month before IN DAM initiation to the month following. No severe adverse events were reported.

**Conclusions:**

After four weeks, IN DAM was a feasible and safe alternative to other routes of administration for patients with severe OUD in HAT. It addressed the needs of individuals with OUD and reduced injection behaviour. More long-term research efforts are needed to systematically assess efficacy of and patient satisfaction with IN DAM.

## Background

Opioid agonist treatment (OAT) is the gold-standard for opioid use disorder (OUD) with a range of available medications used regionally [1]. Sublingual buprenorphine and oral methadone are the most commonly used medications [2]. Other evidence-based and effective OAT medications comprise slow-release oral morphine (SROM), levomethadone, or hydromorphone. Lack of approval impedes access to these medications in most countries, while they are approved and commonly used elsewhere (e.g. Switzerland). However, a wider selection of approved opioids and routes of administration for OAT is crucial for tailoring treatment to the individual needs of patients [3]. This could improve retention in care and treatment satisfaction, while also allowing patients to switch to less harmful routes of administration.

Some patients do not respond to the established forms of OAT and continue high-risk behaviours such as injection use of illicit substances. Specifically, many of these patients seek the euphoric effects provided by fast-acting opioids. To address this need, heroin-assisted treatment (HAT) was introduced as an effective variant of OAT. It is offered in Switzerland, several other European countries, and, sparingly, in Canada [4]. HAT usually consists of the supervised administration of injectable pharmaceutical heroin (diacetylmorphine, DAM). In Switzerland, DAM is available for intravenous (IV) and oral (PO) use with the possibility of take-home doses for up to seven days, and it has also been approved for inhaled use (i.e. smoking) in the Netherlands [5, 6].

HAT providers are facing several clinical challenges. In many parts of Europe, the opioid-dependent population is ageing [3, 7]. Many HAT patients have a long history of IV injecting, which frequently leads to the deterioration of peripheral access veins [8]. They often resort to hazardous inguinal injections [9] or off-label intramuscular (IM) injections [10], which are associated with complications such as muscle tissue indurations, pain, and skin lesions.

Furthermore, patterns of street opioid use in Europe have changed in the past decade. While in 2012, injection was the main route of administration of heroin users entering treatment, inhaling was most common in 2020 [2, 11]. Moreover, the proportion using primarily nasal (IN) heroin (sniffing/snorting) increased from 11 to 25%.

Treatment with PO DAM is the current strategy for Swiss patients for whom conventional OAT is ineffective, or who primarily snort, or are no longer able to inject intravenously. However, the pharmacokinetics of oral DAM differ significantly from other routes of administration such as IV and IN, as well as IM and subcutaneous injecting [12, 13]. PO DAM has a lower morphine-bioavailability and does not result in measurable DAM plasma concentrations as it is rapidly converted into its metabolites [12]. Since DAM passes the blood–brain barrier much faster than morphine due to its lipophilic properties, these pharmacokinetic differences translate into substantial discrepancies in the subjective effects, with PO DAM producing no or only a mild “rush” or euphoria [14].

Many opioid-snorting patients with severe OUD do not enter HAT because the usual prescription of PO DAM is unattractive. A novel route of administration, IN DAM, may be a suitable option to address the needs of these individuals, as well as of patients who do not respond to conventional OAT but are unable/reluctant to inject. Compared to PO DAM, IN DAM has an advantageous pharmacokinetic profile. Following IN administration, maximum plasma levels of DAM (between 4 to 5 min) and its metabolites (between 10 and 45 min) have been measured [15, 16]. Although maximum plasma levels are reached at a much slower rate compared to IV DAM [13], the nasal route could in theory successfully address the need of a subjective “high” (i.e. a rapid onset of action).

IN DAM is currently approved for paediatric pain treatment in the UK, where it has been shown to be safe and effective [17]. Aside from case reports, only one experimental study exists regarding the treatment of OUD [16, 18, 19]. Mitchell et al. investigated the effects of a singular administration of low dose (40 mg) IN DAM in a concentration of 400 mg/ml versus IV DAM in HAT patients. The authors found it to be acceptable, maybe even preferable to injection use, despite a bitter taste. However, DAM doses of 40 mg are of limited clinical relevance, as HAT patients usually require much higher doses.

The IN administration of DAM for the treatment of OUD has not been investigated clinically yet. We report preliminary findings at the four-week follow-up of an open-label cohort study on feasibility and safety of HAT with IN DAM, and the sociodemographic and medical characteristics of patients receiving this treatment form.

## Methods

### Study design

This study is a clinical multicentre observational study of participants in HAT with a duration of three years and planned assessments at baseline, 4, 52, 104 and 156 weeks. All patients interested in receiving IN DAM in HAT were eligible for participation. Consenting participants switched to IN DAM from PO, IM, or IV DAM (treatment as usual) or initiated IN DAM in addition to DAM in other routes of administration. The HAT setting was not altered. Adjustments of DAM dose and route of administration remained an individual decision of patients and prescriber and was independent from study procedures. All patients were followed up regardless of further changes in route of administration of DAM.

### Nasal DAM prescription

**Fig. 1:**
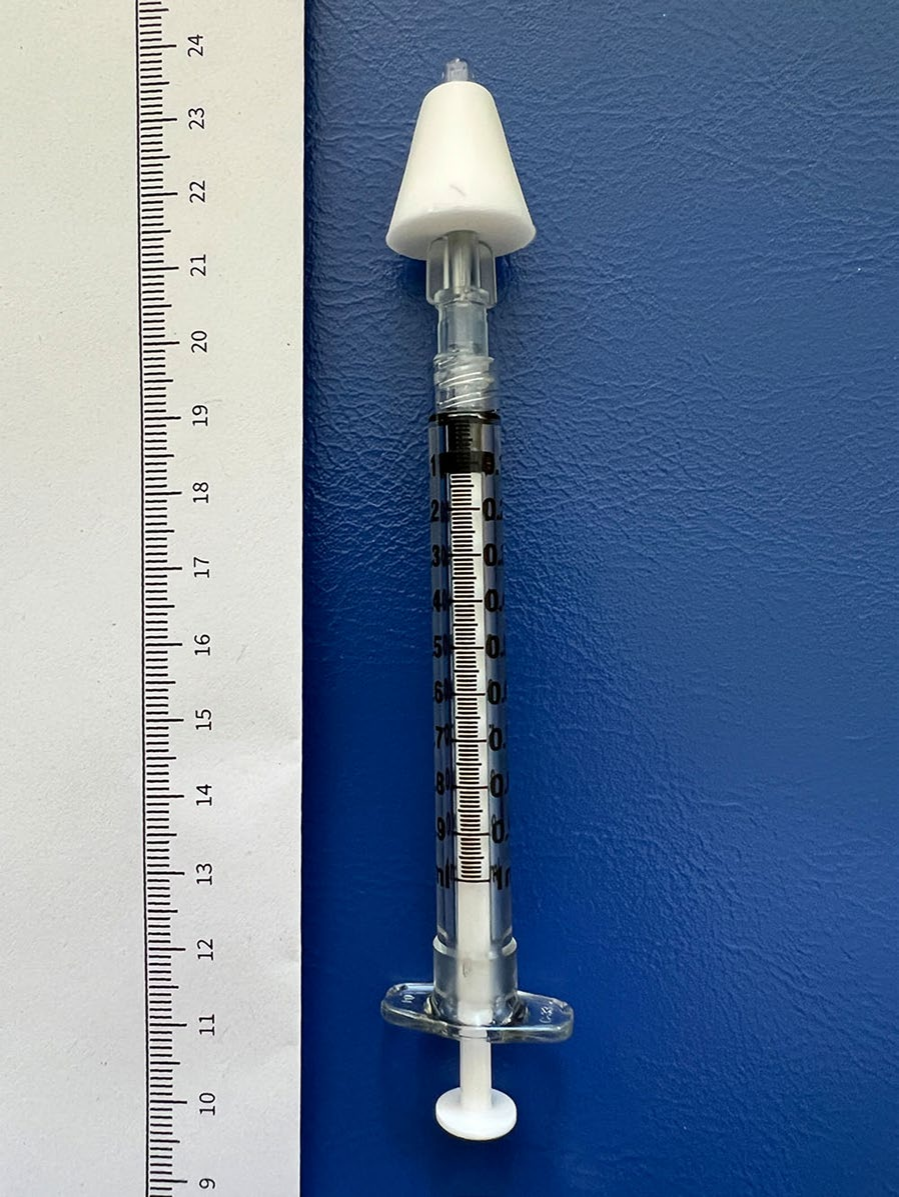
Syringe (1 ml) with screw-on atomiser as used in this study

Initial conversion to nasal doses was calculated using factors of 1.3 for IV DAM and 0.75 for PO DAM derived from available pharmacokinetic data [13, 15, 16, 20], clinical expertise (JS, MV) and currently used factors for conversion of PO and IV DAM in Swiss HAT. Standardised DAM doses for reporting in this paper corresponded to oral morphine equivalent doses and were calculated using a factor of 1 for PO DAM, 2.0 for IV and IM DAM and 1.3 for IN DAM. Sterile DAM solution (100 mg/ml) was used in syringes with screw-on atomisers (Fig. 1). Atomisers were personalised, disinfected following each administration and replaced every seven days.

### Recruitment

Recruitment started in December 2020 and is ongoing. Participants included in this study were recruited from ten Swiss HAT centres (Baar, Basel, Bern, Lausanne, Olten, Reinach, Schaffhausen, Solothurn, Winterthur, Zurich) during routine clinical practice by qualified staff. Inclusions took place after the shared decision to start IN DAM was made by participants and providers, fully independent from study procedures. Informed consent was obtained after participants had been explained the study procedures and had been given the opportunity to ask questions and consider the study. Participants received compensation for time and inconvenience (CHF 40.-) for each of the assessments completed (baseline and week 4 follow-up). Compensation was paid in cash at the end of each assessment, corresponding to a total amount of CHF 80.-. The Federal Office of Public Health provided an exceptional authorisation for off-label IN use of DAM for the time of study conduction. Since study initiation, no applications for IN DAM use outside of the study were made, meaning that all patients receiving IN DAM in Switzerland after December 2020 were successfully recruited for participation.

### Inclusion and exclusion criteria

Inclusion criteria comprised ability to give informed consent, participate in HAT, and wish to receive IN DAM. Entry criteria for HAT in Switzerland include being at least 18 years old, a history of severe opioid dependence of more than two years, having failed at least two conventional treatments for opioid dependence and having documented social or health problems related to opioid dependence. Patients with severe cognitive impairment (e.g. dementia), precluding the completion of the self-report forms/questionnaires, as well as those with insufficient language proficiency were excluded. We excluded four participants from the analyses, because they were already in IN DAM treatment before the study began.

### Assessments

Sociodemographic and medical characteristics were retrieved through electronic medical records (EMRs), interviews, and patient self-report. The last four weeks of DAM prescriptions by route of administration, and both scheduled and realised dispensings were assessed at baseline and four-week follow-up using EMRs.

At baseline, each patient provided the reasons for the switch from their previous route of administration to IN DAM. A series of predefined reasons were available, but participants were also able to expand in their own words. Participants were able to provide as many reasons as they deemed accurate.

Treatment retention for IN DAM was assessed by comparing DAM prescription at baseline to the follow-up assessment at 4-weeks using EMRs. Patients were also asked what their future intentions were regarding the route of administration of their DAM prescriptions.

Given the IN administration, patients’ perceived physical problems relating to their nose and nasal cavity were assessed at baseline and at the four-week follow-up in yes/no form (nasal congestion, runny nose, burning or itching nose, nose pain, epistaxis, and reduced or altered sense of smell).

All assessments were conducted by clinicians working at the respective treatment centre. While filling in self-report forms, participants could ask clarifying questions at any time.

### Statistics

Descriptive statistics were used for sociodemographic characteristics, medical history, reasons for IN DAM, prescription and dispensing history, four-week retention, and nose-related problems. No inferential statistics were conducted. Statistical analyses were conducted with SPSS version 28 (IBM Corp., Armonk, NY, USA).

## Results

Sociodemographic and medical characteristics of the sample (*n* = 52) are presented in Table 1 and Table 2, respectively. The sample is representative of the total population of Swiss HAT patients [21].

**Table 1:** Sociodemographic characteristics of patients receiving IN DAM

Variable (*n*, %)	Sample (*n* = 52)
Age (M, SD)	46.3 (9.9)
Sex	
Female	10 (19.2)
Male	42 (80.8)
Citizenship	
Switzerland	44 (84.6)
Other	8 (15.4)
Living situation	
Private residence	42 (80.8)
Institution*	10 (19.2)
Employment status	
Full time employment	3 (5.8)
Part time employment	4 (7.7)
Unemployed	18 (34.6)
Pension or houseman/wife	24 (46.2)
Other	3 (5.8)
Main source of income	
Wage	6 (11.5)
Wage of partner	2 (3.8)
Pension (invalidity or retirement)	24 (46.2)
Social welfare	19 (36.5)
Other	1 (1.9)

*SD* Standard deviation, *M* Mean, *included assisted living and care home; sample sizes differ due to missing data

**Table 2:** Medical characteristics of patients receiving IN DAM

Variable; *n* (%)	Sample (*n* = 52)
Age of first regular opioid use; *M* (SD)*	20.9 (4.8)
Duration of current HAT episode in years; M (SD)*	8.6 (8.5)
Duration of current HAT episode in years; MD (MIN MAX)*	5.0 (0.0–28.0)
History of non-prescribed IV substance use	45 (86.5)
Non-substance-related psychiatric comorbidities	
None	21 (40.4)
One	13 (25.0)
Two or more	18 (34.6)
Non-opioid-related substance use disorders	
Alcohol use disorder	17 (32.7)
Tobacco use disorder	36 (69.2)
Cocaine use disorder	24 (46.2)
Stimulant use disorder	2 (3.8)
Sedative use disorder	21 (40.4)
Cannabis use disorder	19 (36.5)
Medical comorbidities	
HIV	3 (5.8)
Chronic hepatitis C-infection^1^	6 (11.5)
Liver cirrhosis	4 (7.7)
Chronic obstructive pulmonary disease	6 (11.5)
Diabetes mellitus Type 2	4 (7.7)
Long-term medication	
Benzodiazepines	27 (51.9)
Stimulants	6 (11.5)
Other psychopharmacological medication	24 (46.2)
Other medication (except contraception)	22 (42.3)
Health system utilisation within the past year	
Hospitalisation due to physical illness	12 (23.1)
Emergency room visit	16 (30.8)
Intensive care unit	3 (5.8)
Psychiatric inpatient stay	5 (9.6)
Involuntary psychiatric admission	0 (0)

*SD* Standard deviation, M mean, *MD* Median

^*^sample sizes differ due to missing data; ^1^not including successfully treated HCV-infections

### Reasons for IN DAM

Fifty-one patients provided reasons for switching to or supplementing their prescription with IN DAM. Reasons included preference of snorting over every other route of administration (*n* = 24, 47.1%), currently using IV DAM and having deteriorated peripheral access veins (*n* = 19, 37.3%), a prescription of PO DAM and desire of a stronger effect (*n* = 22, 43.1%), and desire of a less harmful route of administration instead of IV DAM (*n* = 18, 35.3%), IM DAM (*n* = 10, 19.6%), or instead of snorting DAM tablets (*n* = 9, 17.6%). Among those that provided additional details as to the reasons for switching to IN DAM, one patient stated low mood with loss of weight, while another stated cosmetic reasons (injection marks). One patient reported severe induration of muscle tissue due to 20 years of IM DAM use.

### Four-week retention in the IN route of administration

At baseline, 22 patients were receiving only PO DAM (42.3%), eight patients were receiving only IV DAM (15.4%), and three patients were receiving only IM DAM (5.8%). Eight patients were receiving PO DAM and IV DAM (15.4%) and five patients were receiving PO DAM and IM DAM (9.6%). Six patients did not receive DAM at baseline and directly started with IN DAM (11.5%). After four weeks, 47 of 52 patients were still receiving IN DAM (90.4%). Of the five patients that stopped using IN DAM, two had received only PO DAM before study entry, another two had received both IV and PO DAM before inclusion, and one patient had been prescribed both IM and PO DAM. The combinations of various DAM formulations, as well as proportions on additional long-acting/slow-release opioid agonists, are provided in Table 3.

**Table 3:** Prescription DAM combinations and additional long-acting slow-release opioid agonists (*n* = 52)

*n* (%)	Baseline	4-week follow-up
PO and IN	–	23 (44.2)
IN only	–	13 (25.0)
IV and PO and IN	–	7 (13.5)
IV and IN	–	3 (5.8)
PO only	22 (42.3)	2 (3.8)
IV and PO	8 (15.4)	2 (3.8)
IM and IN	–	1 (1.9)
IM and PO	5 (9.6)	1 (1.9)
IV only	8 (15.4)	0 (0.0)
IM only	3 (5.8)	0 (0.0)
No DAM	6 (11.5)	0 (0.0)
Any injected DAM	24 (46.2)	14 (27.0)
Any IN DAM	0 (0.0)	47 (90.4)
Additional methadone or levomethadone	12 (23.1)	11 (21.2)
Additional SROM	27 (51.9)	29 (55.8)

*IN* Intranasal, *IV* Intravenous, *IM* Intramuscular, *PO* Oral, *SROM* Slow-release oral morphine

Patients with an IN DAM prescription attended 81.2% of their scheduled IN dispensings. After four weeks, 90.4% (*n* = 47) of patients wished to continue with their IN DAM prescription in the future.

### Standardised total DAM and IN DAM dose

The total standardised prescribed daily doses of DAM (including IV, IM and PO) ranged from 260 to 3200 mg (M = 1058.0, SD = 626.2, *n* = 46) at baseline and from 208 to 2800 mg (M = 1042.9, SD = 568.9, *n* = 52) in week 4 (including IV, IM, PO and IN).

Total prescribed doses per (repeated) administration of IN DAM ranged from 50 to 700 (M = 274.4, SD = 151.3, *n* = 47). Total prescribed daily doses of IN DAM ranged from 50 to 1400 (M = 415.6, SD = 321.8, *n* = 47).

### DAM injection prescriptions

**Fig. 2:**
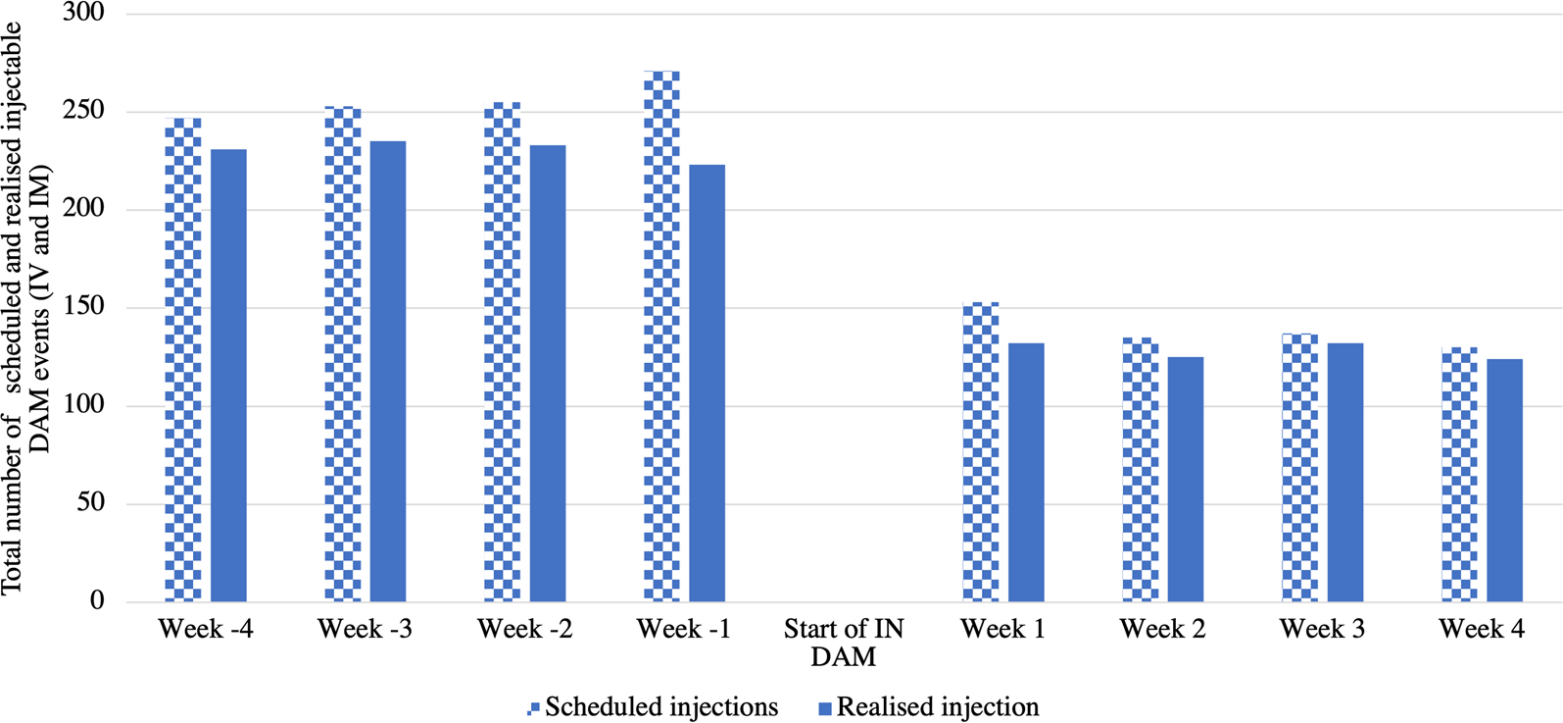
Total scheduled and realised injection events in the sample

In the four weeks prior to study entry, a total of 247 (Week-4), 253 (Week-3), 255 (Week-2), and 271 (Week-1) prescribed DAM injections events were scheduled, including IV and IM (Fig. 2). After initiation of IN DAM, the total number of scheduled prescribed DAM injection events decreased to 153 (Week 1), 135 (Week 2), 137 (Week 3), and 130 (Week 4). This corresponds to a 46.1% decrease from the weekly average in the month before IN DAM initiation (256.5 average scheduled injection events per week) and after (138.3 per week).

Of scheduled prescribed injection events that were actually realised, there were 231 (Week 4), 235 (Week 3), 233 (Week 2), and 223 (Week 1) total realised prescribed DAM injection events, including IV and IM (Fig. 2). After initiation of IN DAM, there were 132 (Week 1), 125 (Week 2), 132 (Week 3), and 124 (Week 4) total realised DAM injection events. Weekly average realised injection events dropped from 230.5 to 128.3 from the month before IN DAM to the month following (44.4% decrease).

### Nose-related adverse events

No serious adverse events occurred. Nose-related symptoms and adverse events were assessed for the previous 4 weeks at baseline and 4-week follow-up (Table 4).

**Table 4:** Self-reported nose-related symptoms and adverse events at baseline (before initiation of IN DAM) and four-week follow-up

*n*, %	Baseline	Week-4 follow-up
Stuffy nose	21 (40.4)	17 (32.7)
Runny nose	29 (55.8)	32 (61.5)
Reduced or altered sense of smell	14 (26.9)	17 (32.7)
Burning or itchy nose	7 (13.5)	5 (9.6)
Nose pain	3 (5.8)	4 (7.7)
Epistaxis	3 (5.8)	4 (7.7)

## Discussion

This is the first study to report on the clinical use of IN DAM in OAT. After a treatment period of four weeks, it appears to be feasible and safe in patients receiving HAT. Retention in this route of administration was high under real-world conditions with 90% of patients continuing to administer IN DAM at the four-week follow-up and the remaining (9.6%) returning to other routes of administration.

A previous study investigated the effects of low-dose IN DAM using a concentration of 400 mg/ml and found it to be acceptable to patients with OUD [16]. We can expand on these findings by using therapeutic doses of IN DAM in a clinical setting, but with a concentration of 100 mg/ml, the solution used for intravenous DAM prescription in Switzerland. Higher concentrations as used by Mitchell et al. would require lower volume of fluid and would therefore be more practical clinically due to the limited absorption capacity of the nasal mucosa. However, the concentration used in this study was chosen for pragmatic reasons. First, DAM in higher concentrations is currently not approved in Switzerland. Second, using a higher concentrated solution for IN DAM could also lead to an increase in the risk of overdose due to potential mix-ups in case of an accidental injection.

The self-reported reasons for switching to IN DAM suggest that it may be particularly suitable for patients with peripheral vein damage due to a long history of injections, preference for snorting over every other route of administration, desire of a stronger effect compared to PO DAM, and desire of a less harmful route of administration compared to IV and IM. Patients in our sample did not provide safety concerns as a reason for switching to IN DAM. However, safety aspects could be a reason for treatment providers to recommend IN DAM to their patients. The nasal route of administration is arguably associated with a better safety profile, including less infections and fewer overdose events, when compared to intravenous or intramuscular routes of administration [22, 23]. Although overdoses and injection-related harms are significantly lower when opioids are used in a treatment setting under supervision, pain and bleeding are still prevalent, and a small risk of infections, seizures and overdose remains [10, 24]. Additionally, substantial decreases in blood oxygenation are observed following opioid injection, which is already a concern among individuals with a history of overdose and among older patients who are at higher risk of pulmonary diseases like COPD [25]. We found that the total number of injection events (both scheduled and realised) per week were nearly halved after the start of IN DAM. Therefore, IN DAM seems suitable to further reduce the risk of long-term DAM use in HAT settings. It may also serve as a transition from injectable to oral DAM and, subsequently, enable switching to oral OAT which allows more patient autonomy and is less cost-intensive.

As well as having an arguably better safety profile compared to IV DAM, IN DAM may also be suitable for some patients for more unique and personal reasons. For instance, one patient stated feeling embarrassed by his scars and injection marks and hoped for his skin to heal following the switch to IN and cessation of IV administration. Patients may therefore be able to avoid stigmatisation associated with injection behaviour, which is a common experience for people who use drugs [24, 26, 27]. All-in-all, IN DAM is attractive to existing as well as new HAT patients. Half of the participants in our study had been attending HAT for more than 5 years, with the longest current HAT episode being 28 years. On the other hand, patients with no history of HAT but who may have been discouraged from receiving previous HAT options were able to enter treatment using this novel route of DAM administration.

Our study provides insights into potential dosing and conversion factors of IN related to IV, IM or PO DAM. Compared to the bioavailability of morphine following PO DAM administration, which has been reported to be around 65% for high doses, the bioavailability following IN DAM administration is likely higher due to the avoidance of the hepatic first-pass effect [28]. This avoidance is mainly limited by the absorption capacity of the nasal mucosa, as exceeding DAM solution may end up being swallowed, resulting in a lower bioavailability compared to IV DAM [29]. We accounted for these differences when converting PO and IV DAM (factor of 1.33 and 0.75, respectively) to IN DAM. Over the four-week study period, the mean DAM dose decreased only slightly, suggesting that the conversion factors we used were clinically reliable and did not lead to withdrawal, only requiring small dose adjustments. However, no data on bioavailability for high dose IN DAM (over 100 mg) is available to date, despite DAM doses of less than 100 mg being rarely used in patients receiving HAT. This underlines the need for further research to explore the specific pharmacokinetic and pharmacodynamic properties of IN DAM solution, in order to inform dosing strategies, which could further improve treatment satisfaction and retention.

In light of the current overdose crisis in North America, our findings have implications for treatment and research. The overdose epidemic is mainly driven by fentanyl and its derivatives. If IN DAM is effective, a similar approach could be adopted by providing nasal fentanyl to individuals who deliberately inject or snort fentanyl [30]. Overall, novel treatment strategies are necessary to meet the changing needs of individuals with OUD, reduce harm and stigma associated with substance use, and move away from a “one size fits all” approach in OAT [3, 31].

### Strengths and limitations

This study has several limitations. We report preliminary data from an ongoing and still recruiting open-label multicentre study and the findings must therefore be interpreted with caution and be seen as preliminary. No inferential statistics were conducted, and we did not recruit matched controls. Therefore, we are unable to test for significant differences in treatment outcomes between patients with and without IN DAM prescriptions. Furthermore, assessments were conducted by clinical staff known to participants. Hence, self-reported data may be subject to social desirability bias. Finally, eligible patients were known by the treatment centre staff and selection bias favouring the stable patients for study inclusion may have had occurred. Since OUD is a chronic medical condition, evaluating continuation/retention of IN DAM over longer periods of time is essential in determining the effectiveness of IN DAM.

The major strengths of this study are that it is a multicentre study across Switzerland, increasing its generalisability. It provides real-world data that make the findings translatable and relevant to clinical practice. Despite its moderate sample size, it is the only clinical study on IN HAT to date.

## Conclusions

IN DAM is a safe and feasible option for HAT. The four-week retention rate for this specific route of administration in HAT was high, no severe adverse events were reported, and the majority of patients indicated an intention to continue using IN DAM moving forward. Overall, IN DAM may be a viable alternative to IV and IM DAM, given the harms associated with injection, as well as to PO DAM, given its more rapid onset of action and associated “rush”. Future studies systematically assessing the acceptability and efficacy of IN DAM over a longer period, among a larger sample and qualitative research assessing the subjective effects of the administrated DAM doses are needed.

## Data Availability

The dataset generated and analysed during the current study are not publicly available due privacy concerns but are available from the corresponding author on reasonable request.

## Abbreviations

DAM: Diacetylmorphine
IN: Intranasal
OAT: Opioid agonist treatment
OUD: Opioid use disorder
SROM: Slow-release oral morphine
HAT: Heroin-assisted treatment
IV: Intravenous
PO: Oral
IM: Intramuscular
EMR: Electronic medical record
